# The time is now for public health to lead the way on addressing financial strain in Canada

**DOI:** 10.17269/s41997-020-00430-2

**Published:** 2020-10-26

**Authors:** Nicole M. Glenn, Candace I. J. Nykiforuk

**Affiliations:** 1 Centre for Healthy Communities, School of Public Health, University of Alberta, 3-035 Edmonton Clinic Health Academy, 11405 – 87 Ave, Edmonton, AB T6G 1C9 Canada; 2 Policy, Location and Access in Community Environments Research Lab, School of Public Health, University of Alberta, 3-300 Edmonton Clinic Health Academy, 11405 – 87 Ave, Edmonton, AB T6G 1C9 Canada

**Keywords:** Populations, underserved, Equity, financial, Health equity, Social determinants of health, Financing, personal, Populations mal desservies, équité financière, équité en santé, déterminants sociaux de la santé, financement individuel

## Abstract

Financial strain was an issue for many Canadians long before the arrival of the global novel coronavirus pandemic in early 2020. However, it has worsened in recent months in relation to the pandemic and public health measures put in place to prevent the spread of COVID-19. Members of underserved groups and people who experience poverty are particularly vulnerable to financial strain and its negative health impacts. As public health professionals, we should be concerned. In this commentary, we discuss the concept of financial strain and its health consequences and highlight how existing research in the area is falling short and why. We suggest next steps to guide research and practice related to financial strain such that it reflects the core values of public health, including equity, life course approaches, and the social determinants of health. This commentary is a call to action for public health researchers and practitioners in Canada to take a more prominent role in shaping the agenda on financial strain to support financial well-being for all.

## Résumé

De nombreux Canadiens subissaient une pression financière bien avant l’arrivée de la pandémie mondiale du nouveau coronavirus au début de 2020. Cette pression s’est toutefois aggravée ces derniers mois en raison de la pandémie et des mesures de santé publique mises en place pour prévenir la propagation de la COVID-19. Les membres des groupes mal desservis et les personnes aux prises avec la pauvreté sont particulièrement vulnérables à la pression financière et à ses effets néfastes sur la santé. En tant que professionnels de la santé publique, nous devrions nous en inquiéter. Dans notre commentaire, nous abordons la notion de pression financière et ses conséquences pour la santé et nous soulignons en quoi et pourquoi la recherche actuelle dans ce domaine n’est pas à la hauteur. Nous proposons des mesures pour orienter la recherche et la pratique liées à la pression financière afin qu’elles reflètent les valeurs fondamentales de la santé publique : l’équité, les approches axées sur la vie entière et les déterminants sociaux de la santé. Ce commentaire est un appel à l’action lancé aux chercheurs et aux praticiens en santé publique du Canada pour qu’ils jouent un plus grand rôle dans la définition des mesures à prendre face à la pression financière afin de favoriser le bien-être financier de tous.

## What is financial strain?

Financial strain is anxiety, distress/stress, worry, or feelings of not coping created by economic events (French and Vigne 2019: p.150). It is commonly measured using survey instruments that ask about perceived ability to cover and cope with current household expenses, such as the InCharge Financial Distress/Financial Well-Being Scale (Prawitz et al. 2006). Financial strain is not the opposite of financial well-being (or financial health/fitness/wellness), which includes objective and subjective assessments of the present and future (French and Vigne 2019; Weida et al. 2020). Instead, it can be understood as part of financial well-being (Netemeyer et al. 2018; Weida et al. 2020).

Financial strain differs from poverty, indebtedness, employment status, and income, which categorize people based on objective measures of financial circumstances (French and Vigne 2019). A person may be under financial pressure according to objective measures (e.g., income) and yet coping well, therefore not experiencing the adverse consequences (e.g., mental health deterioration) of feeling financial strain (French and Vigne 2019). Experiences of poverty or low income have negative consequences and are in need of attention from public health. However, feeling unable to cope financially has its own negative impacts on people’s health (Dijkstra-Kersten et al. 2015; Kahn and Pearlin 2006) that are worthy of consideration.

## Why is financial strain a problem?

Research has demonstrated that stress related to financial circumstances is a key driver of overall well-being (Netemeyer et al. 2018), anxiety, and depression (Dijkstra-Kersten et al. 2015) and can have a detrimental impact on mental (Elwér et al. 2015) and physical health (French and Vigne 2019), independent of objective measures of income (Dijkstra-Kersten et al. 2015; Kahn and Pearlin 2006). Children of adults who experience financial strain are prone to loneliness, depression, and other mental health issues (Dijkstra-Kersten et al. 2015). Taking a life course perspective, chronic financial strain is likely to accumulate over time, resulting in decreased resistance to illness and disease with advancing age (Cruz-Cerdas 2017; Elwér et al. 2015; Kahn and Pearlin 2006). Financial strain is also associated with unhealthy behaviours (e.g., smoking, poor diet) (French and Vigne 2019).

Not having enough money to meet basic needs and pay monthly bills can lead to financial strain, as can unexpected financial shocks (e.g., illness, unplanned pregnancy) (French and Vigne 2019). Consumption patterns, borrowing capacity, savings, assets, and personality traits can all impact financial strain (French and Vigne 2019). Financial strain is also influenced by broader social and economic forces, such as those we are currently facing in Canada and across the globe in relation to the COVID-19 pandemic (Leger 2020).

## Who experiences financial strain?

Like poverty, food insecurity, and low income, financial strain is socially patterned. People belonging to underserved groups, low-income earners, and people who experience poverty often live with continuous cycles and consequences of financial strain due to an inability to meet or go beyond basic needs (French and McKillop 2017; French and Vigne 2019). It is more commonly reported among racialized people (Kahn and Pearlin 2006), mothers (Cruz-Cerdas 2017), and women (Choi et al. 2016; Cruz-Cerdas 2017). Financial stressors can accumulate with other forms of stress resulting from structural oppression (e.g., racism) to amplify the negative health impacts of financial strain (Cruz-Cerdas 2017). That said, people with a steady income and stable housing may also experience financial strain—an unexpected economic shock can make the difference between being able to pay monthly bills or not (French and Vigne 2019). People who experience chronic or severe persistent illness or disease report a high intensity and frequency of financial strain and heightened negative health consequences in relation to that strain (Delgado-Guay et al. 2018).

## How has COVID-19 impacted financial strain?

Prior to the pandemic, people in Canada were already feeling stressed due to their finances (Simpson 2020). This has worsened in recent months: 44% of Canadians reported that COVID-19 has impacted their financial stress (Leger 2020). In addition to an elevated risk of financial strain (French and Vigne 2019), members of underserved groups are at heightened risk of the serious negative impacts of COVID-19 (van Dorn et al. 2020), including the economic consequences such as job loss (Hu 2020) that can lead to financial strain. This has serious equity implications (Hu 2020; van Dorn et al. 2020), which highlights the need for swift and targeted public health action in the area.

## How have public health researchers contributed to understanding financial strain?

Public health researchers have studied the health impacts of financial strain (distress/stress and well-being/health; Dijkstra-Kersten et al. 2015; Elwér et al. 2015); however, they have yet to make significant theoretical contributions to the area (Weida et al. 2020). Although public health researchers and professionals have led the charge on poverty, food and housing security, universal basic income, and other related areas, they have had limited engagement with financial strain specifically or financial health more broadly (Weida et al. 2020). Instead, economists have mostly led the conceptual development of financial strain and the related intervention research (French and Vigne 2019). The same is true of financial well-being (e.g., Prawitz et al. 2006). Unsurprisingly, individual-level interventions, such as financial literacy, have dominated the action on this critical public health issue (Glenn et al., 2020 [unpublished]).

## What are the opportunities and challenges related to focusing on financial strain?

When compared with objective measures, the subjective nature of financial strain can facilitate a deeper understanding of how people are coping financially and their welfare in relation to this (French and McKillop 2017). It can capture differences related to socio-demographic and other historical, social, and contextual factors. Women, for example, report higher levels of stress than men in response to the same (objectively measured) financial circumstances (Choi et al. 2016). That said, focusing on financial strain is not without challenges and potential shortcomings, particularly with regard to the feasibility of its operationalization.

The concept of financial strain is underdeveloped (Delgado-Guay et al. 2018), particularly as it relates to equity and the social determinants of health. There is also a lack of clarity around adjacent concepts and how they relate to each other, particularly financial well-being (health or wellness), which others have noted are not well articulated (Weida et al. 2020). For example, although financial strain or distress/stress are most commonly conceptualized and measured using subjective scales of ability to cover living costs or cope financially (Dijkstra-Kersten et al. 2015), some researchers have relied on objective measures or proxies like ability to raise funds (Elwér et al. 2015).

Upstream interventions targeting social determinants of health like financial strain are inherently challenging. These require political action. Such interventions can be more difficult in times of austerity where resources are sparse and demands are high, as many jurisdictions in Canada are currently facing. Although individual-level interventions are easier to implement and may have a positive impact on participants, they will not address the root causes of financial strain. It is essential that we bring our commitment to the Ottawa Charter (World Health Organization 1986) to target upstream action on financial strain.

## What’s next for public health researchers and professionals to address financial strain?

The current pandemic presents a timely opportunity for learning and meaningful action by public health researchers and professionals on financial strain. We are ideally positioned due to our extensive expertise with respect to related issues such as poverty, food insecurity, and social inclusion. We recommend that public health researchers and professionals target their initial efforts on financial strain in the four areas outlined below. Undertaking these in partnership with practice and significant engagement of people who experience financial strain can ensure the work is relevant and resonates with diverse communities.

1. Develop the concept of financial strain and related measurement tools to align with public health values (e.g., equity, life course perspective). Test and validate measures of financial strain that are meaningful for people across the social spectrum and relate to social, physical, and mental health.
2. Assess policies introduced in response to COVID-19 to reduce financial strain. Consider their impacts on short- and long-term mental, physical, and social health outcomes, across the life course, and in relation to equity and the social determinants of health. Create accessible data sources and shared best practices for their analysis and interpretation.
3. Advocate for continued and increased structural level interventions targeting financial strain (e.g., governmental policy, benefits). Focus on policies and programs that demonstrate a positive impact on equity.
4. Provide support (e.g., training, resources) for community and social services organizations to adopt financial strain measures as part of their program access criteria. Qualification for such programs often relies on income or other objective measures of socio-economic status, which means people in need can fall through the cracks.

As public health researchers and professionals, we support people’s health across the life course while promoting equity and addressing the social determinants of health. The COVID-19 crisis has affirmed our role as trusted sources of health information, programs, and services in Canada and across the globe. The time has come for us to lead when it comes to financial strain.
